# Synergistic Effects of Dietary Protein and Vitamin E Intake on Reducing Metabolic‐Associated Fatty Liver Disease Risk: Insights From NHANES [2017–2023] and Mendelian Randomization Study

**DOI:** 10.1002/fsn3.71026

**Published:** 2025-10-10

**Authors:** Weijie Wu, Jiansheng Chen, Zhiwen Shen, Xiongfeng Lin, Chaoying Fang, Yunzhe Yu, Liqun Liao, Aiming Zeng, Wenjin Ding

**Affiliations:** ^1^ Department of Gastroenterology Fuzhou University Affiliated Provincial Hospital Fuzhou China; ^2^ Department of Digestive Endoscopy Center Fuzhou University Affiliated Provincial Hospital Fuzhou China; ^3^ Department of Gastrointestinal Surgery The First Affiliated Hospital of Fujian Medical University Fuzhou China; ^4^ National Regional Medical Center, Binhai Campus of the First Affiliated Hospital Fujian Medical University Fuzhou China; ^5^ Department of Hepatobiliary and Pancreatic Surgery Taizhou Hospital of Zhejiang Province Affiliated to Wenzhou Medical University Taizhou China; ^6^ Department of Clinical Laboratory Fuzhou University Affiliated Provincial Hospital Fuzhou China; ^7^ Department of Gastroenterology Xin Hua Hospital Affiliated to Shanghai Jiao Tong University School of Medicine Shanghai China

**Keywords:** mendelian randomization, metabolic‐associated fatty liver disease, NHANES, non‐alcoholic fatty liver disease, protein, vitamin E

## Abstract

Nutritional intervention plays a pivotal role in managing metabolic‐associated fatty liver disease (MAFLD), but the combined effect of protein and vitamin E on MAFLD remains indistinct. This study sought to evaluate the synergistic effects of these nutrients on MAFLD. This study enrolled 14,489 participants from the NHANES database. MAFLD was defined by liver fat accumulation and metabolic syndrome components. Multivariable logistic regression, restricted cubic spline modeling, and internal validation were applied to assess the dose–response and joint effects of protein and vitamin E, with gender stratification to explore effect heterogeneity. A Mendelian randomization (MR) approach was utilized to probe the causality between α‐tocopherol and protein intake with MAFLD. Intake levels of protein and vitamin E were significantly reduced in individuals with MAFLD compared to the non‐MAFLD group (All *p* < 0.001). Multivariable logistic regression analysis indicated that combined intake of high protein (> 0.82 g/kg/d) and vitamin E (> 0.140 mg/kg/d) significantly reduced the risk of MAFLD by 72% (OR = 0.280, 95% CI: 0.244–0.320, *p* < 0.001), outperforming the high intake of either nutrient alone; internal validation further confirmed the robustness of the model. Sex‐stratified analysis indicated that elevated intake of protein and vitamin E was associated with a significantly decreased risk of MAFLD in both males and females. MR analysis confirmed that high α‐tocopherol was causally related to a lower risk of MAFLD (IVW OR = 0.871, 95% CI: 0.780–0.973, *p* = 0.015). Although the causality between protein intake and MAFLD was not statistically significant, further analysis indicated that protein intake–associated genetic variants may influence MAFLD through the regulation of carbonic anhydrase 11 expression. This study revealed that combined intake of protein and vitamin E exerts a synergistic protective effect against MAFLD, with gender‐specific effects. It provides translational medical evidence for developing nutrient‐combination‐based therapeutic strategies for MAFLD.

## Graphical abstract

This study reveals that combined intake of protein and vitamin E exerts a synergistic protective effect against MAFLD, with gender‐specific effect. It provides translational medical evidence for developing nutrient‐combination‐based therapeutic strategies for MAFLD.

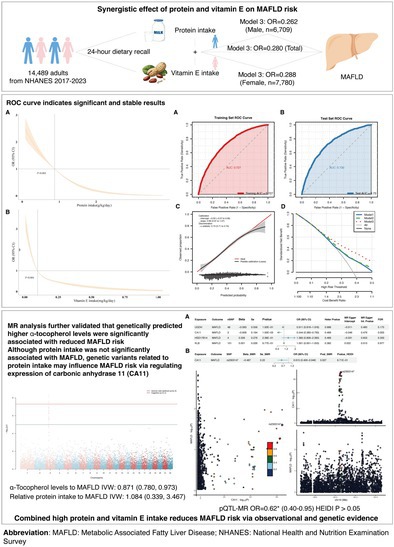

## 1 Introduction

Metabolic‐associated fatty liver disease (MAFLD), previously referred to as non‐alcoholic fatty liver disease, is a chronic and progressive liver condition driven by excess nutrient intake, insulin resistance, and associated metabolic dysfunction in genetically susceptible individuals (Eslam, Newsome, et al. 2020; Eslam, Sarin, et al. 2020). Epidemiological estimates indicate that the global prevalence of MAFLD is approximately 32.4%, with a higher rate in men (39.7%) than in women (25.6%), and this prevalence continues to rise steadily (Lou et al. 2024). MAFLD is not only the most prevalent chronic liver disease globally but is also linked to elevated mortality from malignancies, cardiovascular conditions, and liver‐related complications, thereby posing a significant global public health burden (Riazi et al. 2022).

To date, no targeted pharmacological therapy has been approved for the treatment of MAFLD. Among existing interventions, dietary optimization is considered a cornerstone of MAFLD management (Younossi et al. 2023; Abdallah et al. 2023; Tsompanaki et al. 2023). The three major macronutrients exert distinct effects on MAFLD. Excessive intake of simple carbohydrates, particularly fructose, has been shown to significantly increase hepatic lipid accumulation. Saturated fatty acids and trans fats significantly contribute to hepatic lipid accumulation and inflammation, whereas unsaturated fatty acids confer protective effects by enhancing insulin sensitivity and suppressing inflammatory responses (He et al. 2020). However, the role of dietary protein in MAFLD remains controversial (Tsompanaki et al. 2023). Vitamins are essential for regulating hepatic metabolism, inflammation, and alleviating liver fibrosis induced by a high‐fat diet (Liang et al. 2024; Miao et al. 2024). Although preliminary studies suggest that vitamin E may exert protective effects against MAFLD, most studies have not examined the potential interaction between dietary protein and vitamin E (Nagashimada and Ota 2019; Song et al. 2025).

Compared to the considerable challenge of achieving and sustaining MAFLD remission through weight loss and caloric restriction, modulating specific dietary nutrients may represent a more feasible approach. While preliminary evidence has linked dietary components to the development of MAFLD, there is limited research exploring the combined effects of a “nutrient combination” strategy on MAFLD. Therefore, this study aims to investigate the effect of combined dietary intake of protein and vitamin E on MAFLD using data from the 2017–2023 National Health and Nutrition Examination Survey (NHANES). Subsequently, we conducted an additional Mendelian randomization (MR) analysis to validate the causal relationship between vitamin E, protein, and MAFLD from a genetic perspective, aiming to inform dietary optimization strategies that may reduce the incidence of MAFLD.

## 2 Methods

### 2.1 NHANES

#### 2.1.1 Data Source

**FIGURE 1:**
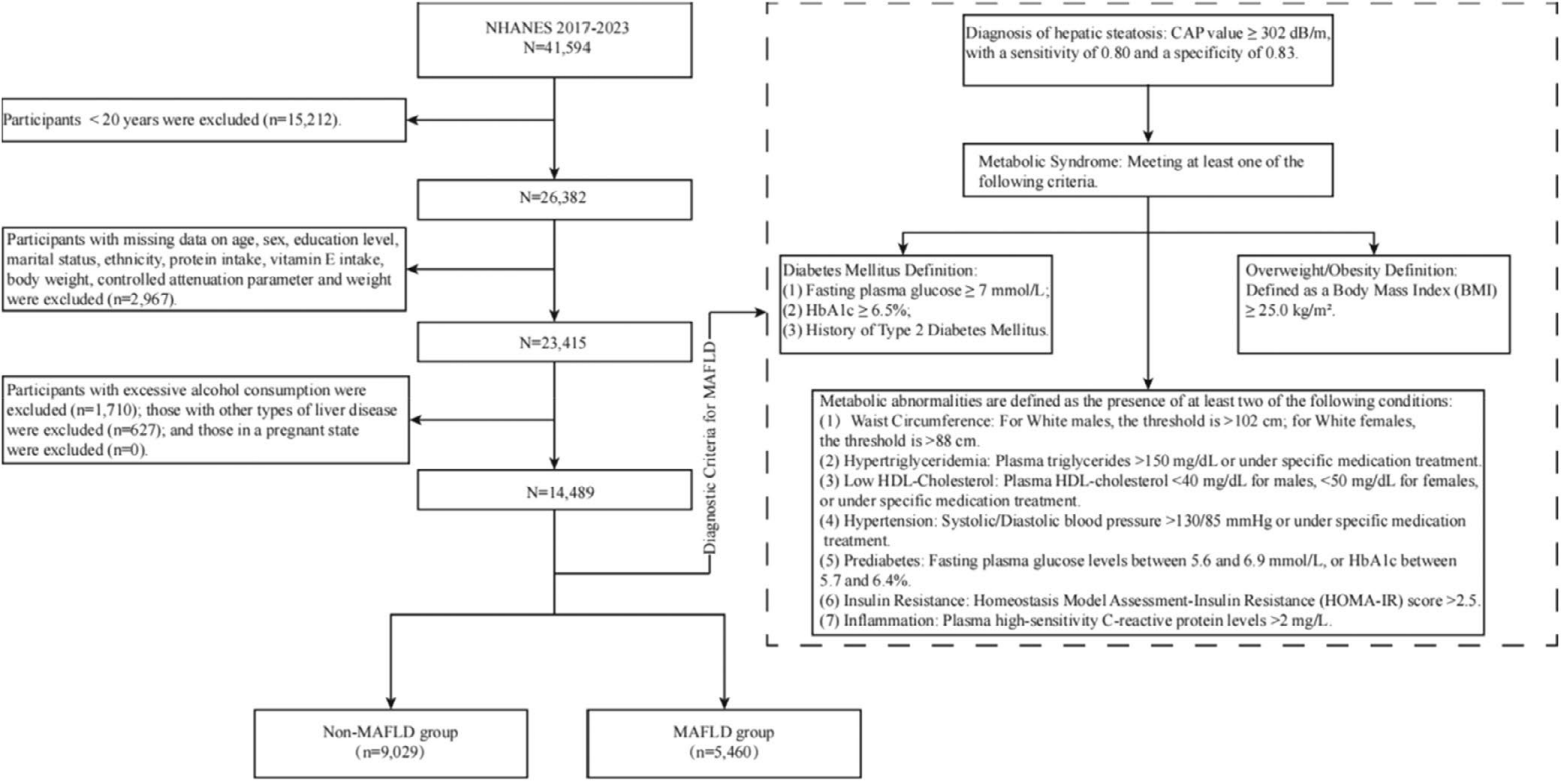
Flow charts for inclusion and exclusion of study samples and diagnostic criteria for MAFLD. BMI, body mass index; CAP, controlled attenuation parameter; HbA1c, hemoglobin A1c; HDL‐C, high‐density lipoprotein cholesterol; HOMA‐IR, Homeostasis Model Assessment of Insulin Resistance (HOMA‐IR); MAFLD, metabolic dysfunction‐associated fatty liver disease; NHANES, National Health and Nutrition Survey.

The data for this study were obtained from NHANES, which is a cross‐sectional survey that employs a nationally representative sampling strategy to evaluate the nutritional and health conditions of the civilian, non‐institutionalized population in the United States. Detailed information regarding the sampling design and data collection procedures is available on the NHANES website (https://www.cdc.gov/nchs/nhanes). Data in NHANES are primarily collected through structured interviews, physical examinations, and laboratory testing, and the survey protocols received ethical approval from the Institutional Review Board of the National Center for Health Statistics (NCHS). Written informed consent was obtained from all participants prior to data collection. During the 2017–2023 survey cycles, NHANES enrolled 41,594 participants in total. After applying inclusion and exclusion criteria, 14,489 participants were deemed eligible and included in the final analysis (Siddiqui et al. 2019; Younossi et al. 2022; Eddowes et al. 2019). A detailed flowchart of the participant selection process is presented in Figure 1.

#### 2.1.2 Measurement of Protein and Vitamin E Intake

Intake of protein and vitamin E was assessed using two 24‐h dietary recall interviews: the initial interview was conducted face‐to‐face at the Mobile Examination Center (MEC); a subsequent interview was administered by telephone within 3 to 10 days after the initial assessment (Ahluwalia et al. 2016). Vitamin E intake encompasses both dietary intake and supplemental intake. The average intake across the two recalls was calculated, and daily protein and vitamin consumption normalized to body weight (g/kg/day) was subsequently analyzed.

#### 2.1.3 Assessment of Covariates

Data were extracted from the questionnaire, physical examination, and laboratory components of NHANES to obtain information on participants' age, sex, educational attainment, body mass index (BMI), marital status, poverty income ratio (PIR), race, physical activity, diabetes, hypertension, smoking status, and comorbidities. Detailed definitions and variable categorizations are provided in Table 1. The confirmation of diabetes was established based on one or more of the following criteria: a fasting plasma glucose concentration ≥ 7.0 mmol/L, an HbA1c level ≥ 6.5%, or a self‐reported documented medical diagnosis of diabetes. Hypertension was identified based on any of the following criteria: diastolic blood pressure ≥ 90 mmHg, systolic blood pressure ≥ 140 mmHg, a documented medical diagnosis of hypertension, or ongoing treatment with prescribed antihypertensive medications. Comorbidities were defined as the presence of cardiovascular disease, kidney disease, or cancer. Demographic covariates with missing values were imputed using predictive mean matching through multiple imputation techniques (Stekhoven and Bühlmann 2012) (Table S1).

**TABLE 1:** Baseline characteristics of MAFLD participants in NHANES 2017–2023.

	Number of participants	Non‐MAFLD	MAFLD	*p* ^a^
	(*n* = 14,489)	(*n* = 9029)	(*n* = 5460)	
Age, (year) (%)				< 0.001
20–40	4205 (29.0%)	3043 (33.7%)	1162 (21.3%)	
41–60	4843 (33.4%)	2762 (30.6%)	2081 (38.1%)	
> 60	5441 (37.6%)	3224 (35.7%)	2217 (40.6%)	
Gender, %				< 0.001
Male	6709 (46.3%)	3913 (43.3%)	2796 (51.2%)	
Female	7780 (53.7%)	5116 (56.7%)	2664 (48.8%)	
Education attainment, %				< 0.001
< High school	865 (5.97%)	483 (5.35%)	382 (7.00%)	
Completed high school	1341 (9.26%)	823 (9.12%)	518 (9.49%)	
> High school	12,283 (84.8%)	7723 (85.5%)	4560 (83.5%)	
Marital status, %				< 0.001
Married/Living with partner	8378 (57.8%)	5047 (55.9%)	3331 (61.0%)	
Widowed/Divorced/Separated/Never married	6111 (42.2%)	3982 (44.1%)	2129 (39.0%)	
Race, %				< 0.001
Mexican American	1522 (10.5%)	715 (7.92%)	807 (14.8%)	
Non‐Hispanic White	5949 (41.1%)	3604 (39.9%)	2345 (42.9%)	
Non‐Hispanic Black	3447 (23.8%)	2389 (26.5%)	1058 (19.4%)	
Other Hispanic	1398 (9.65%)	866 (9.59%)	532 (9.74%)	
Other Race	2173 (15.0%)	1455 (16.1%)	718 (13.2%)	
PIR^b^, %				< 0.001
Low income	2223 (15.3%)	1392 (15.4%)	831 (15.2%)	
Middle income	9671 (66.7%)	5933 (65.7%)	3738 (68.5%)	
High income	2595 (17.9%)	1704 (18.9%)	891 (16.3%)	
BMI (kg/m^2^), SD	30.3 (7.51)	27.6 (6.11)	34.7 (7.53)	< 0.001
Physical activity, %				0.026
Inactive	7001 (48.3%)	4279 (47.4%)	2722 (49.9%)	
Moderate	4055 (28.0%)	2556 (28.3%)	1499 (27.5%)	
Vigorous	520 (3.59%)	339 (3.75%)	181 (3.32%)	
Both moderate and vigorous	2913 (20.1%)	1855 (20.5%)	1058 (19.4%)	
Smoking status, %				< 0.001
Former	3566 (24.6%)	2020 (22.4%)	1546 (28.3%)	
Current	10,767 (74.3%)	6885 (76.3%)	3882 (71.1%)	
Never	156 (1.08%)	124 (1.37%)	32 (0.59%)	
Diabetes, %				< 0.001
No	11,768 (81.2%)	8006 (88.7%)	3762 (68.9%)	
Yes	2721 (18.8%)	1023 (11.3%)	1698 (31.1%)	
Hypertension, %				< 0.001
No	8086 (55.8%)	5649 (62.6%)	2437 (44.6%)	
Yes	6403 (44.2%)	3380 (37.4%)	3023 (55.4%)	
Comorbidity^c^, %				< 0.001
No	11,182 (77.2%)	7099 (78.6%)	4083 (74.8%)	
Yes	3307 (22.8%)	1930 (21.4%)	1377 (25.2%)	
Protein intake (g/kg/day), SD	0.95 (0.45)	1.02 (0.49)	0.82 (0.36)	< 0.001
Vitamin E intake (mg/kg/day), SD	0.12 (0.10)	0.14 (0.11)	0.10 (0.07)	< 0.001

Abbreviations: BMI, body mass index (kg/m^2^); MAFLD, metabolic dysfuction‐associated fatty liver disease; NHANES, National Health and Nutrition Examination Surveys; PIR, poverty income ratio; SD, standard deviation.

^a^ *p*‐Value were computed using weighted linear regression analyses or the Wilcoxon rank‐sum test for continuous variables, and the weighted chi‐square test was employed for categorical variables.

^b^ A PIR of ≤ 1 is classified as low income, a PIR between 1 and 4.98 as middle income, and a PIR > 4.98 as high income.

^c^ The presence of any one of heart disease, kidney disease, or cancer is considered a comorbid condition.

### 2.2 Statistical Analysis

Descriptive statistics were used to present baseline participant characteristics, with continuous variables expressed as means ± standard errors, and categorical variables reported as frequencies alongside weighted proportions. Comparisons of continuous variables across groups were assessed using linear regression and the Wilcoxon rank‐sum tests, as appropriate. Differences in categorical variables were assessed using chi‐square tests. The relationships between protein (g/kg/day) and vitamin E intake (mg/kg/day) with the risk of MAFLD were examined using multivariable logistic regression models. The magnitude of these associations was expressed as odds ratios (ORs) with corresponding 95% confidence intervals (CIs). In addition, the Bonferroni method was applied for multiple testing correction, with *p* values below the corrected threshold regarded as statistically significant (Assennato and Bruzzi 2002). Dose–response relationships between protein and vitamin E intake and the risk of MAFLD were assessed using restricted cubic spline (RCS) regression models. Participants were further stratified by quartiles, with Q1 and Q2 defined as low intake and Q3 and Q4 as high intake, resulting in four dietary patterns: low protein and vitamin E intake, low protein and high vitamin E intake, high protein and low vitamin E intake, and high protein and vitamin E intake. The differences in MAFLD prevalence among these groups were subsequently assessed. Three models were fitted to test the robustness of our results: Model 1 was unadjusted; Model 2 was adjusted to account for age stratification; Model 3 included additional adjustments for sex, marital status, race, PIR, educational attainment, physical activity, smoking status, diabetes, hypertension, and comorbidities based on Model 2. Moreover, the study performed a Wald test for multiplicative interaction between protein and vitamin E intake groups to evaluate the association between their synergistic effects and MAFLD (Wald 1943).

To assess model robustness, internal validation was conducted using the NHANES dataset, with the sample was randomly partitioned into a training set (70%) and a validation set (30%). A multivariable logistic regression model was developed in the training set and subsequently applied to the validation set for predictive assessment. Model discrimination and robustness were evaluated using the receiver operating characteristic (ROC) curve and the corresponding area under the curve (AUC), along with Harrell's C‐index and its 95% CI. Calibration was assessed through bootstrap resampling (1000 iterations), with calibration curves generated and the model intercept (ideal = 0) and slope (ideal = 1) reported. The relationship between predicted and observed probabilities was further examined using a Loess‐smoothed calibration curve. Finally, decision curve analysis was performed to evaluate the net clinical benefit of the model across a range of risk thresholds (Efron 1979).

### 2.3 Mendelian Randomization

#### 2.3.1 Data Source

Mendelian randomization is a statistical approach that employs genetic variants—specifically single nucleotide polymorphisms (SNPs)—as instrumental variables (IVs) to infer potential causal links between risk factors and health outcomes. Genotypes are randomly inherited from parents to offspring and remain stable regardless of age or lifestyle, thereby minimizing confounding and reverse causation, which are often present in observational studies, and enhancing the reliability of causal inference (Emdin et al. 2017). The genetic instruments for relative protein intake and α‐tocopherol levels (the most active form of vitamin E) were derived from genome‐wide association studies (GWAS) (https://gwas.mrcieu.ac.uk/datasets) based on the UK Biobank dataset, which includes over 330,000 individuals of European ancestry (Meddens et al. 2021; Panyard et al. 2021). Additionally, the study utilized a GWAS dataset for MAFLD, comprising 770,180 control individuals and 8434 diagnosed cases (Ghodsian et al. 2021) (Table S2).

#### 2.3.2 Instrumental Variables Selection

The identification of IVs was guided by the three fundamental assumptions of MR. (1) The genetic variants must exhibit a strong association with the exposure of interest: SNPs meeting the significance threshold (*p* < 5 × 10^−8^) were retained for analysis. Due to the limited number of IVs for vitamin E, the threshold was relaxed to *p* < 1 × 10^−6^. (2) The genetic variants must be independent of confounding factors: To ensure independence between SNPs, a stringent linkage disequilibrium (LD) threshold of *R* ^2^ < 0.001 was applied. Additionally, a clumping window of 10,000 kilobases was set to minimize pleiotropic effects. (3) The selected genetic variants must influence the outcome exclusively through their effect on the exposure of interest: SNPs with potential associations with the outcome were identified and removed using the LDtrait tool (https://ldlink.nih.gov/?tab=home) to ensure compliance with the exclusion restriction criterion. Additionally, for SNPs missing in the outcome dataset, proxy SNPs exhibiting strong linkage disequilibrium (LD > 0.8) were considered. In cases where no proxy SNP was available, the original SNP was excluded from further analysis. SNPs with palindromic alleles were also excluded. The *F*‐statistic served as a key indicator of instrument strength. To mitigate potential bias from weak instrument variables in Mendelian randomization (MR), SNPs with an *F*‐statistic below 10 were excluded from the analysis. Following these quality control procedures, a set of SNPs suitable for MR analysis was selected (Emdin et al. 2017) (Tables S3 and S4).

### 2.4 Two‐Sample Mendelian Randomization Analysis

This study performed two‐sample MR analyses using multiple complementary approaches, including inverse variance weighting (IVW), MR‐Egger, and Mendelian Randomization Pleiotropy RESidual Sum and Outlier (MR‐PRESSO), and weighted median (WM) methods. The causal inference primarily relied on the random‐effects IVW method, which assumes that the MR estimates across SNPs are normally distributed, thereby providing robust causal estimates and better accommodating potential heterogeneity. The WM method provides unbiased estimates when at least 50% of SNPs satisfy the three core MR assumptions, despite its lower statistical efficiency compared to IVW. MR‐Egger regression introduces an intercept term to the IVW model, facilitating causal inference even in the presence of horizontal pleiotropy. The regression slope estimates the causal effect of α‐tocopherol and protein on MAFLD. However, due to its lower statistical power and wider CIs, the MR‐Egger intercept is primarily used for sensitivity analysis to assess the potential influence of horizontal pleiotropy (Bowden et al. 2016, 2015). The MR‐PRESSO approach was additionally employed to identify and correct for horizontal pleiotropy. To evaluate heterogeneity, Cochran's *Q* statistic was utilized to determine whether there was significant variation in the effects of individual SNPs. In addition, a leave‐one‐out sensitivity analysis was performed by omitting each SNP and repeating the MR analysis to determine whether any dominant SNPs were driving the conclusions. OR was used to represent the effect size in the analysis (Verbanck et al. 2018; Hemani et al. 2018). Furthermore, the two‐sample Mendelian randomization approach is less susceptible to false‐positive bias compared to single‐sample methods, as it incorporates outcome data from an independent cohort (Burgess et al. 2016).

We further employed the summary data‐based Mendelian randomization (SMR) method to investigate the potential effects of genetic variants associated with protein intake traits on MAFLD risk. SMR is an analytical framework that integrates publicly available expression quantitative trait locus (eQTL) and protein quantitative trait locus (pQTL) data with GWAS summary statistics, leveraging SNPs strongly associated with gene expression or protein abundance as instrumental variables to examine potential causal or colocalization relationships between molecular traits and target phenotypes (Wu et al. 2018; Mai et al. 2023). In this study, we first identified regulatory genes associated with SNPs related to relative protein intake, extracted SNPs reaching genome‐wide significance (*p* < 5 × 10^−8^), and then matched them with the corresponding pQTL dataset (https://download.decode.is/form/folder/proteomics) (Table S3). The pQTL data were subsequently integrated with GWAS summary statistics for MAFLD, and SMR analyses were performed using SMR software (version 1.3.1). To distinguish true causal effects from LD‐driven colocalization signals, the Heterogeneity in Dependent Instruments (HEIDI) test was performed following SMR analysis. The HEIDI test evaluates heterogeneity across multiple SNP instruments, with *p* HEIDI ≥ 0.05 indicating that the observed association is more likely attributable to a single causal variant rather than LD‐induced pleiotropy. Associations meeting the criteria *p* SMR < 0.05 and *p* HEIDI > 0.05 were considered to have high confidence for causal or colocalization relationships (Ferkingstad et al. 2021).

Unless stated otherwise, statistical procedures were conducted in R software (http://www.r‐project.org; version 4.2.0), primarily utilizing the “survey” package (version 4.4.2) and the “Two Sample MR” package (version 0.6.9). Several supplementary R packages were also used to support the analysis.

## 3 Results

### 3.1 Demographic and Clinical Characteristics of Participants

A total of 14,489 eligible individuals were ultimately included in the study and categorized into two cohorts according to MAFLD status: the MAFLD group (*n* = 5460) and the non‐MAFLD group (*n* = 9029). The two groups exhibited significant differences across various baseline variables, including age, sex, BMI, race, educational attainment, marital status, PIR, physical activity, smoking status, prevalence of diabetes and hypertension, and comorbidities (all *p* < 0.05). Notably, lower intake levels of protein [MAFLD vs. non‐MAFLD: (0.82 ± 0.36) vs. (1.02 ± 0.49), *p* < 0.001] and vitamin E [MAFLD vs. non‐MAFLD: (0.10 ± 0.07) vs. (0.14 ± 0.11), *p* < 0.001] were observed in the MAFLD group (Table 1).

### 3.2 Association Between Protein and Vitamin E Intake and MAFLD

**FIGURE 2:**
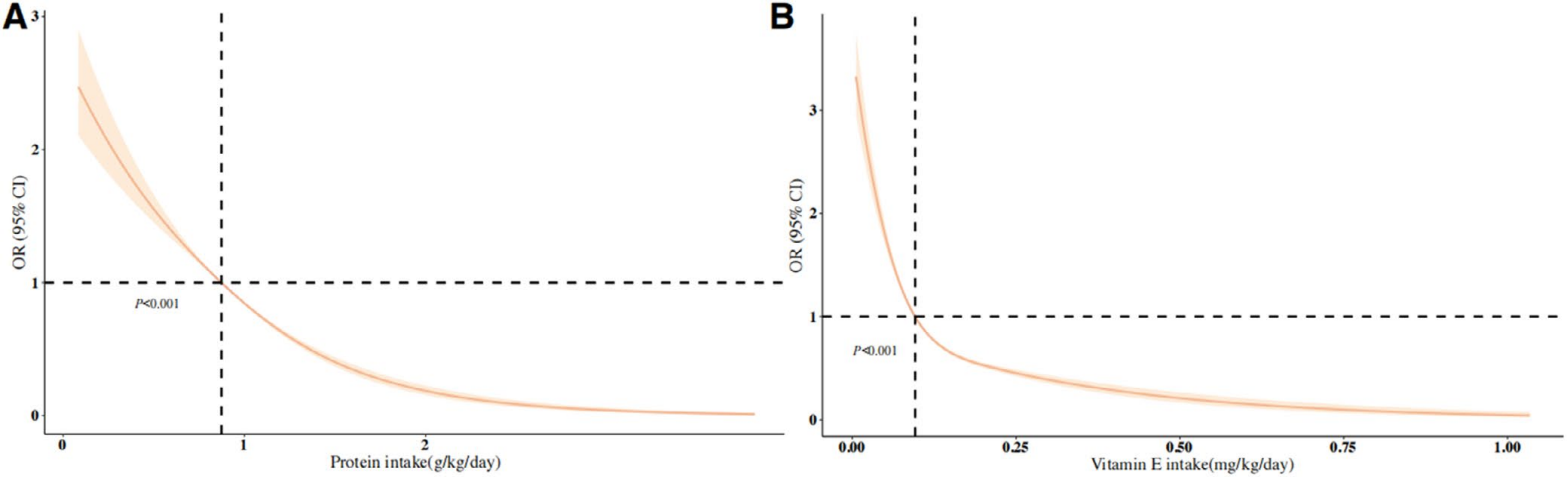
Association of protein (A) and vitamin E (B) intake with MAFLD risk based on restricted cubic spline (RCS) analysis. The solid line represents the estimated OR, while the shaded region indicates the 95% confidence interval. The reference point is set at the median protein intake (0.874 g/kg/day) and vitamin E intake (0.096 mg/kg/day), marked by the vertical dashed line. The horizontal dashed line at OR = 1 represents the null effect.

The logistic regression model presents the ORs and associated trends for MAFLD across stratified intakes of protein and vitamin E (Table 2). In the protein intake stratification, participants in the highest quartile of protein consumption consistently exhibited the lowest prevalence of MAFLD relative to those in the lowest quartile across all models [Model 3, OR: 0.239, 95% CI: (0.203, 0.282), *p* < 0.001]. A consistent association was identified for vitamin E, where individuals in the highest intake quartile exhibited the lowest prevalence of MAFLD [Model 3, OR: 0.251, 95% CI: (0.205, 0.306), *p* < 0.001]. Using the RCS model, we further evaluated the relationship between protein and vitamin E intake and the risk of MAFLD. The results indicated a general decline in MAFLD risk as protein and vitamin E intake increased (*p* < 0.001) (Figure 2).

**TABLE 2:** Association of MAFLD with protein intake and vitamin E intake.

MAFLD	Total participants	MAFLD participants	Model 1 OR (95% CI)	*p*‐Value (Bonferroni, *m* = 6)	Model 2 OR (95% CI)	*p*‐Value (Bonferroni, *m* = 6)	Model 3 OR (95% CI)	*p*‐Value (Bonferroni, *m* = 6)
Protein intake (g/kg/day)								
Q1 (≤ 0.657)	4019	1989	Ref		Ref		Ref	
Q2 (0.657–0.905)	3694	1584	0.728 (0.621, 0.851)	< 0.001	0.720 (0.615, 0.844)	< 0.001	0.718 (0.605, 0.852)	< 0.001
Q3 (0.905–1.178)	3226	1124	0.488 (0.401, 0.593)	< 0.001	0.482 (0.399, 0.582)	< 0.001	0.487 (0.399, 0.596)	< 0.001
Q4 (> 1.178)	3550	763	0.232 (0.198, 0.271)	< 0.001	0.240 (0.206, 0.280)	< 0.001	0.239 (0.203, 0.282)	< 0.001
Vitamin E intake (mg/kg/day)								
Q1 (≤ 0.0652)	3901	1949	Ref		Ref		Ref	
Q2 (0.0652–0.0979)	3587	1531	0.753 (0.618, 0.918)	0.006	0.731 (0.598, 0.894)	0.003	0.722 (0.593, 0.877)	0.001
Q3 (0.0979–0.150)	3628	1214	0.446 (0.379, 0.525)	< 0.001	0.430 (0.363, 0.510)	< 0.001	0.447 (0.371, 0.537)	< 0.001
Q4 (> 0.150)	3373	766	0.246 (0.202, 0.300)	< 0.001	0.239 (0.197, 0.289)	< 0.001	0.251 (0.205, 0.306)	< 0.001

*Note:* Model 1 = unadjusted. Model 2 = Adjusted for age (20–40, 40–60 and > 60 years). Model 3 = Model2 puls additional adjustment for sex, education level, marital status, race, poverty income ratio, physical activity, smoking status, diabetes, hypertension, comorbidity. Q1–Q4 represent quartiles of protein intake (g/kg/day) or vitamin E intake (mg/kg/day), with Q1 being the lowest and Q4 the highest intake group. Statistical significance was assessed using Bonferroni correction for multiple comparisons (significance threshold: 0.05/6).

Abbreviations: 95% CI, 95% confidence interval; MAFLD, metabolic dysfunction‐associated fatty liver disease; OR, odds ratio.

### 3.3 Relationship Between Combined Intake and MAFLD

To explore the effect of combined protein and vitamin E intake on MAFLD, participants were classified into quartiles based on intake levels: Q1 and Q2 representing low intake, and Q3 and Q4 representing high intake. To further investigate the effect of combined protein and vitamin E intake on MAFLD, we categorized participants based on their intake levels into quartiles, with Q1 and Q2 defined as the low intake group and Q3 and Q4 as the high intake group. Participants were categorized into four distinct groups according to protein and vitamin E intake levels: low protein and vitamin E intake group (*n* = 5631), low protein and high vitamin E intake group (*n* = 2082), high protein and low vitamin E intake group (*n* = 1863), and high protein and vitamin E intake group (*n* = 4913) (Table 3). Subgroup analysis indicated that, compared to the low protein and low vitamin E group, higher intake of either or both nutrients was associated with a significantly lower prevalence of MAFLD (*p* < 0.001). Furthermore, the prevalence of MAFLD generally decreased with increasing intake of both vitamin E and protein. This is consistent with the dose–response relationship between protein and vitamin E intake and the prevalence of MAFLD as presented in Table 2. Table S5 presents the association analysis of the interaction variable combining protein and vitamin E intake with MAFLD risk. Among the 16 possible combinations, most showed statistically significant protective effects, with the high‐protein and high–vitamin E intake combination (Q4*Q4) exhibiting the strongest protective effect [Model 3: OR = 0.141, 95% CI: (0.110, 0.180), *p* < 0.001]. The overall Wald test for the interaction variable reached statistical significance, confirming its substantial contribution to the model and highlighting a synergistic effect of the two nutrients.

**TABLE 3:** Baseline characteristics based on protein and vitamin E intake.

	Number of participants	Low protein and vitamin E intake^d^	Low protein and high vitamin E intake	High protein and low vitamin E intake	High protein and vitamin E intake	*p* ^a^
	(*n* = 14,489)	(*n* = 5631)	(*n* = 2082)	(*n* = 1863)	(*n* = 4913)	
MAFLD, %						< 0.001
No	9029 (62.3%)	2843 (50.5%)	1297 (62.3%)	1166 (62.6%)	3723 (75.8%)	
Yes	5460 (37.7%)	2788 (49.5%)	785 (37.7%)	697 (37.4%)	1190 (24.2%)	
Age, (year) (%)						< 0.001
20–40	4205 (29.0%)	1554 (27.6%)	476 (22.9%)	556 (29.8%)	1619 (33.0%)	
41–60	4843 (33.4%)	1877 (33.3%)	748 (35.9%)	634 (34.0%)	1584 (32.2%)	
> 60	5441 (37.6%)	2200 (39.1%)	858 (41.2%)	673 (36.1%)	1710 (34.8%)	
Gender, %						< 0.001
Male	6709 (46.3%)	2320 (41.2%)	789 (37.9%)	1087 (58.3%)	2513 (51.2%)	
Female	7780 (53.7%)	3311 (58.8%)	1293 (62.1%)	776 (41.7%)	2400 (48.8%)	
Education attainment, %						< 0.001
< High school	865 (5.97%)	383 (6.80%)	77 (3.70%)	163 (8.75%)	242 (4.93%)	
Completed high school	1341 (9.26%)	628 (11.2%)	131 (6.29%)	193 (10.4%)	389 (7.92%)	
> High school	12,283 (84.8%)	4620 (82.0%)	1874 (90.0%)	1507 (80.9%)	4282 (87.2%)	
Marital status, %						< 0.001
Married/Living with partner	8378 (57.8%)	3028 (53.8%)	1227 (58.9%)	1181 (63.4%)	2942 (59.9%)	
Widowed/Divorced/Separated/Never married	6111 (42.2%)	2603 (46.2%)	855 (41.1%)	682 (36.6%)	1971 (40.1%)	
Race, %						< 0.001
Mexican American	1522 (10.5%)	509 (9.04%)	170 (8.17%)	287 (15.4%)	556 (11.3%)	
Non‐Hispanic White	5949 (41.1%)	2226 (39.5%)	959 (46.1%)	761 (40.8%)	2003 (40.8%)	
Non‐Hispanic Black	3447 (23.8%)	1695 (30.1%)	504 (24.2%)	318 (17.1%)	930 (18.9%)	
Other Hispanic	1398 (9.65%)	546 (9.70%)	163 (7.83%)	221 (11.9%)	468 (9.53%)	
Other Race	2173 (15.0%)	655 (11.6%)	286 (13.7%)	276 (14.8%)	956 (19.5%)	
PIR^b^, %						< 0.001
Low income	2223 (15.3%)	1066 (18.9%)	230 (11.0%)	275 (14.8%)	652 (13.3%)	
Middle income	9671 (66.7%)	3840 (68.2%)	1412 (67.8%)	1270 (68.2%)	3149 (64.1%)	
High income	2595 (17.9%)	725 (12.9%)	440 (21.1%)	318 (17.1%)	1112 (22.6%)	
BMI (kg/m^2^), SD	30.3 (7.51)	34.0 (8.13)	30.9 (6.89)	28.7 (5.62)	26.5 (5.24)	< 0.001
Physical activity, %						< 0.001
Inactive	7001 (48.3%)	2846 (50.5%)	1011 (48.6%)	941 (50.5%)	2203 (44.8%)	
Moderate	4055 (28.0%)	1479 (26.3%)	639 (30.7%)	485 (26.0%)	1452 (29.6%)	
Vigorous	520 (3.59%)	212 (3.76%)	59 (2.83%)	72 (3.86%)	177 (3.60%)	
Both moderate and vigorous	2913 (20.1%)	1094 (19.4%)	373 (17.9%)	365 (19.6%)	1081 (22.0%)	
Smoking status, %						< 0.001
Former	3566 (24.6%)	1400 (24.9%)	579 (27.8%)	499 (26.8%)	1088 (22.1%)	
Current	10,767 (74.3%)	4191 (74.4%)	1481 (71.1%)	1345 (72.2%)	3750 (76.3%)	
Never	156 (1.08%)	40 (0.71%)	22 (1.06%)	19 (1.02%)	75 (1.53%)	
Diabetes						< 0.001
No	11,768 (81.2%)	4280 (76.0%)	1704 (81.8%)	1513 (81.2%)	4271 (86.9%)	
Yes	2721 (18.8%)	1351 (24.0%)	378 (18.2%)	350 (18.8%)	642 (13.1%)	
Hypertension, %						< 0.001
No	8086 (55.8%)	2768 (49.2%)	1118 (53.7%)	1068 (57.3%)	3132 (63.7%)	
Yes	6403 (44.2%)	2863 (50.8%)	964 (46.3%)	795 (42.7%)	1781 (36.3%)	
Comorbidity^c^, %						< 0.001
No	11,182 (77.2%)	4213 (74.8%)	1562 (75.0%)	1444 (77.5%)	3963 (80.7%)	
Yes	3307 (22.8%)	1418 (25.2%)	520 (25.0%)	419 (22.5%)	950 (19.3%)	

^a^ p‐Value were computed using weighted linear regression analyses or the Wilcoxon rank‐sum test for continuous variables, and the weighted chi‐square test was employed for categorical variables.

^b^ A PIR of ≤ 1 is classified as low income, a PIR between 1 and 4.98 as middle income, and a PIR > 4.98 as high income.

^c^ The presence of any one of heart disease, kidney disease, or cancer is considered a comorbid condition.

^d^ Protein intake (g/kg/day) was categorized into quartiles: quartile 1 (Q1, ≤ 0.657), quartile 2 (Q2, 0.657–0.905), quartile 3 (Q3, 0.905–1.178), and quartile 4 (Q4, > 1.178). Similarly, vitamin E intake (mg/kg/day) was divided into quartiles: quartile 1 (Q1, ≤ 0.0652), quartile 2 (Q2, 0.0652–0.0979), quartile 3 (Q3, 0.0979–0.150), and quartile 4 (Q4, > 0.150). For analysis, Q1 and Q2 were classified as the low intake group, while Q3 and Q4 were categorized as the high intake group. Other abbreviate consistent with Table 1.

**FIGURE 3:**
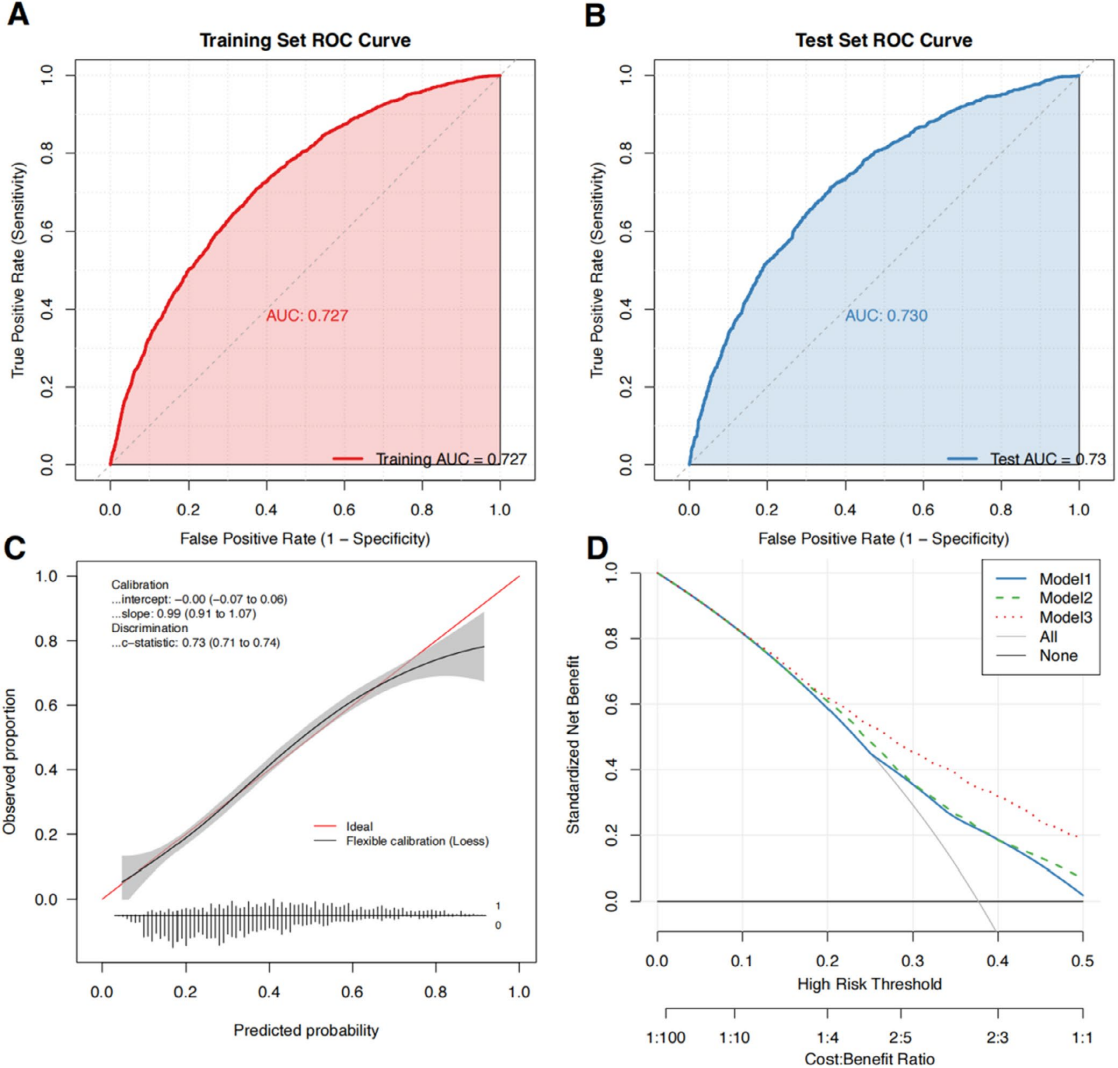
Sensitivity analyses within the NHANES dataset: (A) receiver operating characteristic (ROC) curve of the training set; (B) ROC curve of the test set; (C) calibration curve; (D) decision curve analysis.

To further assess the robustness of the model, internal validation was conducted using the NHANES dataset, which was randomly divided into a training set (70%) and a test set (30%). The AUC of the model was 0.727 in the training set and 0.730 in the validation set (C‐index = 0.73, 95% CI: 0.71–0.74), indicating stable discriminative ability. The calibration curve demonstrated strong agreement between the predicted probabilities and the observed outcomes, the calibration intercept ≈0 (95% CI: −0.07–0.06), and the calibration slope = 0.99 (95% CI: 0.91–1.07), both approximating the ideal values, suggesting the model exhibited neither systematic bias nor overfitting. Decision curve analysis further showed that Model 3 provided greater net benefit across a broad range of threshold probabilities (Figure 3).

### 3.4 Association Between Combined Intake and MAFLD Stratified by Sex

To investigate the relationship between combined intake of protein and vitamin E and MAFLD, logistic regression analyses were conducted, with comparisons made across models with varying adjustments. In the unadjusted model (Model 1), compared to the low protein and vitamin E intake group, which served as the reference group (Ref), a significantly lower risk of MAFLD was observed in the groups with the low protein and high vitamin E intake group [OR = 0.522, 95% CI: (0.440, 0.617), *p* < 0.001], the high protein and low vitamin E intake group [OR = 0.562, 95% CI: (0.462, 0.682), *p* < 0.001], and the high protein and vitamin E intake group [OR = 0.269, 95% CI: (0.233, 0.310), *p* < 0.001], with the trend becoming more pronounced as vitamin E and protein intake increased. Following age adjustment in Model 2, the relationship remained consistent, with the high protein and vitamin E intake group exhibiting an OR of 0.270 (95% CI: 0.237–0.307, *p* < 0.001). With additional adjustments for confounding factors, including sex, educational attainment, and others (Model 3), the OR for the high protein and vitamin E intake group remained at 0.280 [95% CI: (0.244, 0.320), *p* < 0.001], supporting the robustness of this association (Table 4).

**TABLE 4:** Association between gender‐stratified varying levels protein with vitamin E intake and the risk of MAFLD.

MAFLD	Total participants	MAFLD participants	Model 1 OR (95% CI)	*p*‐Value (Bonferroni, *m* = 9)	Model 2 OR (95% CI)	*p*‐Value (Bonferroni, *m* = 9)	Model 3 OR (95% CI)	*p*‐Value (Bonferroni, *m* = 9)
Total								
Low protein and vitamin E intake	5631	2788	Ref		Ref		Ref	
Low protein and high vitamin E intake	2082	785	0.522 (0.440, 0.617)	< 0.001	0.491 (0.414, 0.581)	< 0.001	0.511 (0.427, 0.613)	< 0.001
High protein and low vitamin E intake	1863	697	0.562 (0.462, 0.682)	< 0.001	0.561 (0.463, 0.679)	< 0.001	0.543 (0.449, 0.656)	< 0.001
High protein and vitamin E intake	4913	1190	0.269 (0.233, 0.310)	< 0.001	0.270 (0.237, 0.307)	< 0.001	0.280 (0.244, 0.320)	< 0.001
Male								
Low protein and vitamin E intake	2320	1274	Ref		Ref		Ref	
Low protein and high vitamin E intake	789	334	0.565 (0.410, 0.780)	< 0.001	0.542 (0.393, 0.747)	< 0.001	0.600 (0.439, 0.820)	0.002
High protein and low vitamin E intake	1087	470	0.645 (0.477, 0.871)	0.005	0.666 (0.497, 0.892)	0.008	0.647 (0.471, 0.874)	0.006
High protein and vitamin E intake	2513	718	0.267 (0.212, 0.337)	< 0.001	0.264 (0.211, 0.331)	< 0.001	0.262 (0.197, 0.343)	< 0.001
Female								
Low protein and vitamin E intake	3311	1514	Ref		Ref		Ref	
Low protein and high vitamin E intake	1293	451	0.497 (0.399, 0.618)	< 0.001	0.454 (0.363, 0.568)	< 0.001	0.496 (0.393, 0.616)	< 0.001
High protein and low vitamin E intake	776	227	0.392 (0.285, 0.539)	< 0.001	0.365 (0.266, 0.501)	< 0.001	0.361 (0.270, 0.483)	< 0.001
High protein and vitamin E intake	2400	472	0.250 (0.199, 0.313)	< 0.001	0.250 (0.201, 0.311)	< 0.001	0.288 (0.226, 0.364)	< 0.001

*Note:* Model 1 = unadjusted. Model 2 = Adjusted for age (20–40, 40–60 and > 60 years). Model 3 = Model2 puls additional adjustment for the sex, education level, marital status, race, poverty income ratio, physical activity, smoking status, diabetes, hypertension, comorbidity. Statistical significance was assessed using Bonferroni correction for multiple comparisons (significance threshold: 0.05/9).

Abbreviations: 95% CI, 95% confidence interval; MAFLD, metabolic dysfunction‐associated fatty liver disease; OR, odds ratio.

In the sex‐stratified analysis, compared with the reference group (Ref), a significant inverse relationship between high intake of both protein and vitamin E and the risk of MAFLD was observed in both males [Model 3, OR = 0.262, 95% CI: (0.197, 0.343), *p* < 0.001] and females [Model 3, OR = 0.288, 95% CI: (0.226, 0.364), *p* < 0.001]. Moreover, the protective effect of high protein and low vitamin E intake was more pronounced in females [Model 3, OR = 0.361, 95% CI: (0.270, 0.483), *p* < 0.001] than in males [Model 3, OR = 0.647, 95% CI: (0.471, 0.874), *p* = 0.006], suggesting that women may be more responsive to the protective effects of a high protein and low vitamin E intake compared to men (Table 4). After adjustment for multiple testing, the associations described above retained statistical significance.

### 3.5 Mendelian Randomization Analysis

A MR analysis was conducted to evaluate the potential causal relationships between α‐tocopherol levels, relative protein intake, and the risk of MAFLD. A total of 17 IVs strongly associated with α‐tocopherol levels and 2 related to relative protein intake were obtained from the GWAS database (Tables 5, S2–S4).

**TABLE 5:** Mendelian randomization analysis from vitamin E levels and relative protein intake to MAFLD.

Exposure	Outcome	MR methods	nSNP	*p*‐Value (Bonferroni, *m* = 2)	OR (95% CI)	Heterogeneity test		MR‐Egger pleiotropy test		MR‐PRESSO	
						*q*	*p*	Intercept	*p*	Outliers	*p*
α‐Tocopherol levels	MAFLD	Inverse variance weighted	17	0.015	0.871 (0.780, 0.973)	14.378	0.571	0.022	0.268	0	0.554
α‐Tocopherol levels	MAFLD	Weighted median	17	0.046	0.847 (0.720, 0.997)						
α‐Tocopherol levels	MAFLD	MR Egger	17	0.041	0.781 (0.629, 0.970)						
Relative protein intake	MAFLD	Inverse variance weighted	2	0.891	1.084 (0.339, 3.467)	0.203	0.652		—	—	—

*Note:* Statistical significance was assessed using Bonferroni correction for multiple comparisons (significance threshold: 0.05/2).

Abbreviations: MAFLD, metabolic dysfunction‐associated fatty liver disease; MR, Mendelian randomization; MR‐PRESSO, Mendelian Randomization Pleiotropy RESidual Sum and Outlier; OR, odds ratio; SNP, single nucleotide polymorphism.

In the MR analysis of α‐tocopherol levels and MAFLD, the IVW method demonstrated a significant inverse association between α‐tocopherol levels and the risk of MAFLD [OR = 0.871, 95% CI: (0.780, 0.973), *p* = 0.015], which remained statistically significant after applying the Bonferroni‐corrected threshold (*α* = 0.025). Although the WM method [OR = 0.847, 95% CI: (0.720, 0.997), *p* = 0.046] and MR‐Egger regression did not remain significant after correction for multiple comparisons, the direction of effect was concordant with the IVW findings. Both the MR‐PRESSO global tests and MR‐Egger intercept indicated an absence of horizontal pleiotropy (*p* > 0.05). However, the leave‐one‐out sensitivity analysis revealed that removing rs116455686 significantly affected the estimated association between α‐tocopherol levels and MAFLD. Nevertheless, the overall association still suggested a protective effect of α‐tocopherol against MAFLD (Figure S1). In contrast, no significant association between relative protein intake and MAFLD was observed [IVW OR = 1.084, 95% CI: (0.339, 3.467), *p* = 0.891].

**FIGURE 4:**
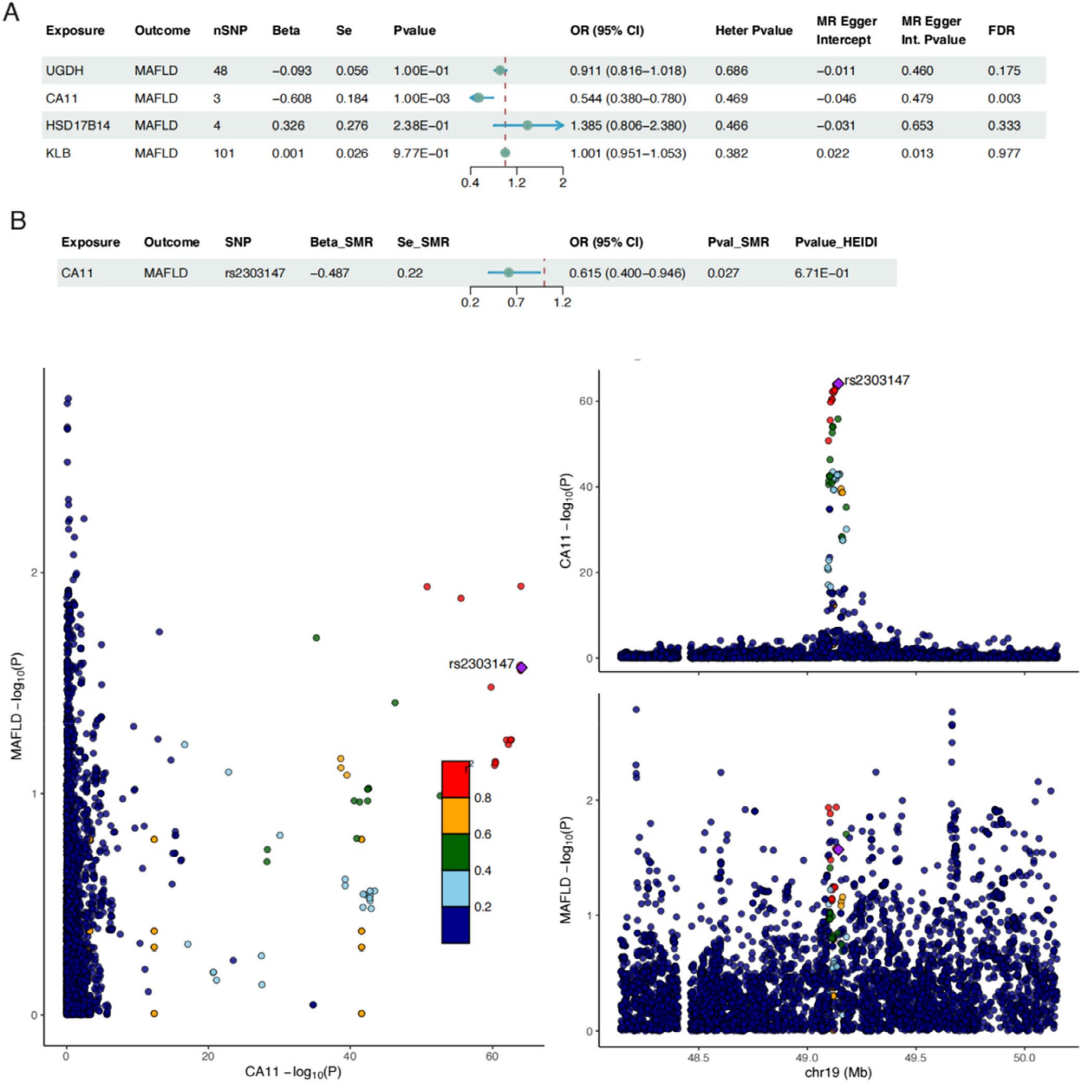
Mendelian randomization analysis of genetically predicted protein levels in relation to MAFLD. (A) pQTL‐MR analysis. (B) SMR analysis of *CA11* with MAFLD and Manhattan plot.

Considering that overall relative protein intake might obscure the effects of certain genetic signals, we further integrated regulatory genes of protein intake–associated SNPs with pQTL data and performed pQTL‐MR and SMR analyses. The results revealed that CA11 protein levels, among protein intake–related genes, were significantly inversely associated with MAFLD risk [OR = 0.615, 95% CI: (0.400, 0.946), *p* = 0.027], and colocalization analysis supported that rs2303147 simultaneously regulates CA11 levels and MAFLD risk (Figure 4).

## 4 Discussion

To our knowledge, this is the first NHANES‐based study to combine large‐scale observational and genetic data to assess the impact of combined nutrient intake on MAFLD. The study assessed protein and vitamin E intake among 14,489 participants between 2017 and 2023. Based on preliminary findings showing differing levels of protein and vitamin E intake between MAFLD and non‐MAFLD groups, it further confirmed a dose–response association between protein and vitamin E exposure levels and the prevalence of MAFLD after multiple model adjustments. Specifically, increased intake of either nutrient was consistently linked to a reduced prevalence of MAFLD, while their combined intake demonstrated a stronger synergistic protective effect. The study further investigated sex‐specific differences in the associations between nutrient intake and MAFLD. These results offer important implications for formulating nutritional interventions to reduce the risk of MAFLD.

As a lipid‐soluble antioxidant, vitamin E has demonstrated protective effects in non‐alcoholic fatty liver disease models to scavenge free radicals, mitigate oxidative damage in hepatocyte endoplasmic reticulum and mitochondria, and suppress the secretion of pro‐inflammatory cytokines including IL‐6 and TNF‐α (Nagashimada and Ota 2019; Demirel‐Yalciner et al. 2021). Moreover, Sanyal's clinical trial demonstrated that vitamin E supplementation led to significant clinical improvements in patients with nonalcoholic steatohepatitis (Sanyal et al. 2010). Studies have suggested that dietary protein intake stimulates glucagon secretion and elevates intestinal levels of glucagon‐like peptide‐1 (GLP‐1) in response to high‐protein consumption. GLP‐1 exerts its effects by activating its receptor, which enhances insulin signaling, reduces hepatocellular lipotoxicity and oxidative stress, and thereby alleviates hepatic steatosis (Ichikawa et al. 2023; van der Klaauw et al. 2013; Yabut and Drucker 2023). In addition, in individuals with diabetes and obesity, increased protein intake has been shown to inhibit hepatic expression of the transcription factor SREBP‐1c, thereby downregulating lipogenic enzymes and limiting hepatic lipid production (Torres et al. 2006). Vitamin E and branched‐chain amino acids (BCAAs) derived from dietary protein activate the AMPK signaling pathway, resulting in phosphorylation of acetyl‐CoA carboxylase and subsequent suppression of SREBP‐1c expression. Vitamin E also inhibits NF‐κB‐mediated inflammatory responses and, in conjunction with cysteine‐rich proteins, promotes glutathione synthesis to enhance the production of anti‐inflammatory mediators. Collectively, the evidence supports a synergistic role of dietary protein and vitamin E in mitigating hepatic lipotoxicity (Demirel‐Yalciner et al. 2021; Chen et al. 2023; Zhang et al. 2024). This finding plausibly explains the dose–response relationship observed in our study between protein and vitamin E intake and the prevalence of MAFLD. Additionally, participants in the high protein and vitamin E intake group exhibited more favorable BMI, greater physical activity levels, and reduced rates of diabetes and hypertension relative to those in the low intake group. These findings suggest that higher intake of protein and vitamin E is associated with a more favorable metabolic profile. Notably, while increasing either protein or vitamin E intake alone can reduce the risk of MAFLD, their combined consumption provides superior protection, suggesting a synergistic effect in which vitamin E may serve as a key enhancer. Therefore, in the management of MAFLD, it may be essential to emphasize adequate vitamin E intake, establish a dual‐threshold system based on dosage and proportion, and enhance the monitoring of α‐tocopherol levels as a biomarker for evaluating the efficacy of nutritional interventions. Furthermore, prospective randomized controlled trials should be conducted to confirm the efficacy of combined vitamin E and protein interventions, providing stronger evidence for MAFLD prevention and management.

In light of the markedly higher prevalence of MAFLD among males compared to females, this study investigated the potential sex‐specific effects of optimizing protein and vitamin E intake (Lou et al. 2024). Overall, a high intake of either protein or vitamin E was consistently linked to a lower risk of MAFLD in both males and females, with the combination of high protein and high vitamin E intake demonstrating the most pronounced protective effect. The ORs were lower in females, suggesting that the protective effects of high protein or high vitamin E intake might be more pronounced in women. However, after adjustment for confounding variables in Model 3, the protective effect of the combined high protein and high vitamin E intake against MAFLD appeared stronger in men than in women. This difference may be attributed to a greater baseline prevalence of adverse health factors, such as smoking and hypertension, among men prior to adjustment (Mauvais‐Jarvis et al. 2020). Females appeared more responsive to the combination of high protein and low vitamin E intake, which may be attributed to the multifactorial compensatory effects of estrogen, including its antioxidant, anti‐inflammatory, and lipid metabolism‐modulating properties. In contrast, males are more reliant on vitamin E due to the pro‐oxidative, pro‐inflammatory environment and visceral fat distribution driven by androgens (Camporez et al. 2019; Kasarinaite et al. 2023). This finding underscores the need for sex‐specific nutritional strategies, wherein women may compensate for antioxidant insufficiency by optimizing protein quality, while men should ensure sufficient vitamin E intake to maximize the efficacy of interventions. Future research should incorporate variations in protein sources and dynamic hormonal changes to further elucidate the underlying mechanisms. Clarifying sex‐specific protective effects mediated by hormonal interactions could provide a scientific basis for tailored nutritional strategies.

Building upon the findings of the cross‐sectional analysis, we further employed MR analysis to genetically validate the potential causal relationships between both exposures and MAFLD. The MR analysis indicated that α‐tocopherol, the primary bioactive form of vitamin E, is likely to exert a protective effect against MAFLD, consistent with findings from previous retrospective studies (Nagashimada and Ota 2019; Scorletti et al. 2022). However, MR analysis did not reveal any statistically significant causal link between relative protein intake and MAFLD. This finding contrasts with the associations observed in the NHANES dataset; the number of significant SNPs currently available is indeed limited, which may introduce potential bias. Subsequent SMR and colocalization analyses indicate that certain genetic variants associated with protein intake may influence MAFLD risk indirectly by modulating CA11 protein levels rather than through direct effects of total protein consumption. Previous evidence has also indicated that CA11 is involved in adipogenesis and fatty acid biosynthetic metabolic processes (Supuran 2022). Protein intake in NHANES includes both animal‐ and plant‐based sources; however, the genetic instruments employed in the MR analysis, derived from GWAS results, cannot differentiate between specific sources or protein quality and may not precisely represent the exposure variable as defined in NHANES. Although MR did not confirm an independent causal effect of protein, the discrepancy with observational findings provides important scientific insights. It suggests that the protective effect of protein may depend on its source and synergistic interactions with nutrients such as vitamin E. Moreover, there may be dynamic thresholds of metabolic demand for protein at different stages of MAFLD, which are challenging to capture using genetic instruments. Future studies should integrate proteomics with SNP–environment interaction analyses to elucidate the genetic regulatory basis of “high‐quality protein.” Additionally, among study participants, the prevalence of MAFLD was lower in current smokers than in former smokers. Notably, the development of MAFLD results from long‐term metabolic dysfunction. Compared with former smokers, who generally have a longer smoking history, current smokers may not have reached the threshold of exposure necessary to induce substantial hepatic injury.

This study is the first to demonstrate, in a large population, the synergistic protective effect of combined protein and vitamin E intake in reducing the risk of MAFLD. It addresses the limitations of previous studies that focused on individual nutrients. The study also proposes sex‐specific nutritional strategies and provides novel evidence to support revisions of dietary guidelines. Moreover, by integrating clinical data from NHANES with genetic validation through MR, the study methodologically minimizes the potential impact of reverse causality. This enhances the robustness of the study's conclusions. Nevertheless, several limitations should be acknowledged. First, this study assessed participants' protein and vitamin E intake using two 24‐h dietary recall (24HDR) records collected at different time points in the NHANES database. While this approach is both practical and representative for large population‐based studies, it may be subject to recall bias and inter‐individual dietary variation. Short‐term dietary records may not accurately reflect long‐term dietary patterns. Although major confounding factors such as age, diabetes, and smoking were adjusted for to the greatest extent possible, the influence of unmeasured confounders—such as the potential role of the gut microbiome—cannot be entirely ruled out. The samples used in the NHANES and MR analyses were drawn from different populations, which may have introduced potential biases into the findings. Owing to the limited number of SNPs associated with protein and vitamin E, multivariable MR analysis was not conducted in this study; future studies should expand GWAS sample sizes to validate more refined causal relationships. Despite the robust performance in internal validation, which provides strong evidence for the reliability of the model, future validation in other independent cohorts will further confirm its generalizability, as there is currently insufficient data to support external validation. Although the NHANES analysis suggested that high protein intake was protective against MAFLD, the MR analysis failed to confirm a causal link. The risk of developing MAFLD may depend on the specific types or sources of dietary protein. The effects of animal‐based and plant‐based proteins on MAFLD may differ. However, due to the lack of detailed classification of protein sources in the NHANES dataset, subgroup analysis based on protein types could not be performed. Therefore, future studies will conduct stratified analyses to evaluate the effects of different protein sources and the combined intake of protein and vitamin E on MAFLD. This may offer a novel preventive paradigm for MAFLD—which currently lacks effective pharmacological treatments—based on the synergistic effects of dietary components. It may also promote a shift in MAFLD prevention and treatment from single‐nutrient interventions to personalized dietary strategies.

## 5 Conclusion

This study found that increased combined intake of protein and vitamin E is significantly linked to a reduced risk of MAFLD and supports sex‐specific dietary optimization strategies. Given the current lack of effective pharmacological treatments for MAFLD, increasing the combined intake of protein and vitamin E may serve as a promising nutritional intervention. This study provides evidence supporting a “nutrient combination” strategy for MAFLD intervention, which could contribute to the shift in dietary guidelines towards multi‐target, synergistic interventions.

## Author Contributions

**Weijie Wu:** conceptualization (equal), funding acquisition (equal), investigation (equal), writing – original draft (lead). **Jiansheng Chen:** conceptualization (lead), methodology (equal), software (equal), writing – original draft (lead). **Zhiwen Shen:** conceptualization (equal), methodology (equal), software (equal), validation (equal), visualization (equal), writing – review and editing (equal). **Xiongfeng Lin:** data curation (equal), investigation (equal), methodology (equal), writing – review and editing (equal). **Chaoying Fang:** data curation (equal), formal analysis (equal), methodology (equal), writing – review and editing (equal). **Yunzhe Yu:** data curation (equal), formal analysis (equal), investigation (equal), methodology (equal), writing – review and editing (equal). **Liqun Liao:** data curation (equal), formal analysis (equal), methodology (equal), validation (equal), writing – review and editing (equal). **Aiming Zeng:** conceptualization (equal), project administration (equal), resources (equal), supervision (lead), writing – review and editing (equal). **Wenjin Ding:** data curation (equal), formal analysis (equal), investigation (equal), project administration (equal), writing – original draft (equal).

## Ethics Statement

This study made use of publicly accessible data from the National Health and Nutrition Examination Survey (NHANES), which is conducted by the National Center for Health Statistics (NCHS), a division of the Centers for Disease Control and Prevention (CDC). All data are de‐identified and accessible to the public; therefore, this study was exempt from institutional review board approval and complies with the ethical standards of the Declaration of Helsinki.

## Conflicts of Interest

The authors declare no conflicts of interest.

## Supporting information


**Figure S1:** Scatter plot of causal effects of vitamin E (A) and relative protein intake (B) on MAFLD. Leave‐one‐out sensitivity analysis for causal effects of vitamin E on MAFLD (C). MAFLD, metabolic dysfuction‐associated fatty liver disease.

**Table S1:** Missingness and treatment of each covariate.
**Table S2:** Phenotype descriptions and distributions.
**Table S3:** Instrumental variables and associated genes for α‐tocopherol levels and relative protein intake.
**Table S4:** Detailed information on SNPs from alpha‐tocopherol levels and relative protein intake to MAFLD.
**Table S5:** Association of combined protein and vitamin E intake quartiles with MAFLD.

## Data Availability

The datasets supporting the conclusions of this article are available in [the National Health and Nutrition Examination Survey (NHANES), conducted by the National Center for Health Statistics (NCHS), Centers for Disease Control and Prevention (CDC)] repository, [https://wwwn.cdc.gov/nchs/nhanes/search/default.aspx]. All R scripts for statistical analysis and visualization have been uploaded to a public GitHub repository (https://github.com/Chen‐Jiansheng/Available‐data‐and‐code.git).
