# Supplementary figures and images for: Synergistic Effects of Dietary Protein and Vitamin E Intake on Reducing Metabolic‐Associated Fatty Liver Disease Risk: Insights From NHANES [2017–2023] and Mendelian Randomization Study
Source: Food Sci Nutr. 2025 Oct 10;13(10):e71026. doi: 10.1002/fsn3.71026 (PMC12511786; doi:10.1002/fsn3.71026)

**Supplementary_figureS1**
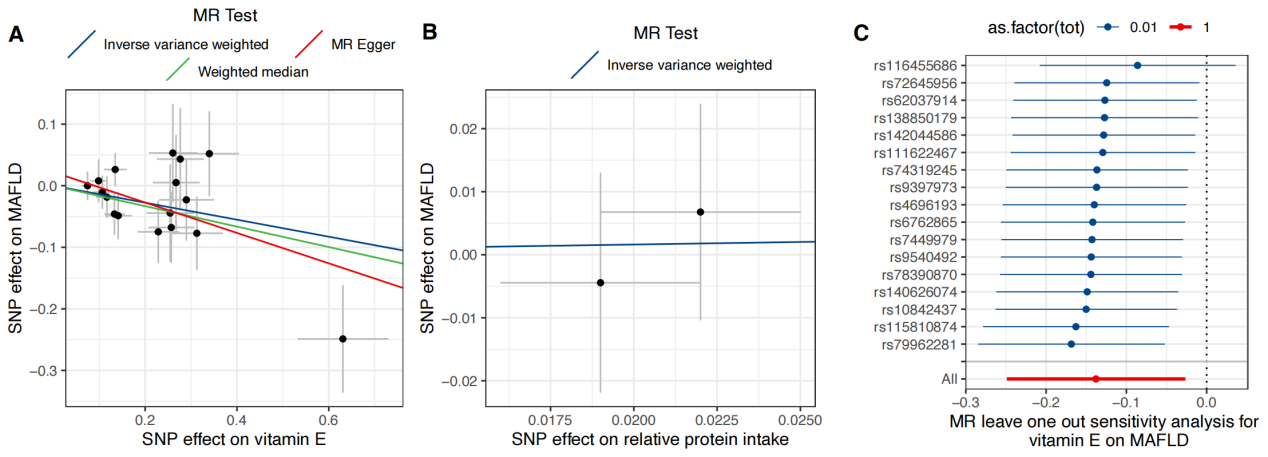

Supplement: Supplementary file 1 — Figure S1: Scatter plot of causal effects of vitamin E (A) and relative protein intake (B) on MAFLD. Leave‐one‐out sensitivity analysis for causal effects of vitamin E on MAFLD (C). MAFLD, metabolic dysfuction‐associated fatty liver disease. [file FSN3-13-e71026-s002.docx]
